# Translation of Viral mRNA without Active eIF2: The Case of Picornaviruses

**DOI:** 10.1371/journal.pone.0022230

**Published:** 2011-07-14

**Authors:** Ewelina Welnowska, Miguel Angel Sanz, Natalia Redondo, Luis Carrasco

**Affiliations:** Centro de Biología Molecular “Severo Ochoa” (CSIC-UAM), Universidad Autónoma de Madrid, Madrid, Spain; Institut Pasteur, France

## Abstract

Previous work by several laboratories has established that translation of picornavirus RNA requires active eIF2α for translation in cell free systems or after transfection in culture cells. Strikingly, we have found that encephalomyocarditis virus protein synthesis at late infection times is resistant to inhibitors that induce the phosphorylation of eIF2α whereas translation of encephalomyocarditis virus early during infection is blocked upon inactivation of eIF2α by phosphorylation induced by arsenite. The presence of this compound during the first hour of infection leads to a delay in the appearance of late protein synthesis in encephalomyocarditis virus-infected cells. Depletion of eIF2α also provokes a delay in the kinetics of encephalomyocarditis virus protein synthesis, whereas at late times the levels of viral translation are similar in control or eIF2α-depleted HeLa cells. Immunofluorescence analysis reveals that eIF2α, contrary to eIF4GI, does not colocalize with ribosomes or with encephalomyocarditis virus 3D polymerase. Taken together, these findings support the novel idea that eIF2 is not involved in the translation of encephalomyocarditis virus RNA during late infection. Moreover, other picornaviruses such as foot-and-mouth disease virus, mengovirus and poliovirus do not require active eIF2α when maximal viral translation is taking place. Therefore, translation of picornavirus RNA may exhibit a dual mechanism as regards the participation of eIF2. This factor would be necessary to translate the input genomic RNA, but after viral RNA replication, the mechanism of viral RNA translation switches to one independent of eIF2.

## Introduction

The genome of picornaviruses comprises a molecule of single-stranded RNA of positive polarity that also acts as the only viral mRNA that is translated in infected cells [1]. Upon binding of the virion to its receptor, the naked viral particles deliver the ssRNA molecule to the cytoplasm, where it is recognized and translated by the cellular protein synthesizing machinery [2]. This early viral translation is followed by RNA replication giving rise to large amounts of RNA molecules of positive polarity, some of which may serve as new mRNAs to direct the massive synthesis of viral proteins during the late phase of infection [3], [4], [5]. This late viral translation is accompanied by a profound inhibition of cellular protein synthesis. The mechanism by which picornavirus mRNA is translated has been analyzed from the early days of research on eukaryotic protein synthesis. In fact, encephalomyocarditis virus (EMCV) RNA was the first viral mRNA to be translated in a mammalian cell free system [6]. Shortly afterwards, the requirements for different eIFs were investigated, revealing that eIF2 was necessary for EMCV mRNA translation [7]. Since then, all experiments with picornavirus mRNAs have provided overwhelming evidence for requirement of eIF2 for the initiation of picornavirus protein synthesis in cell free systems and in culture cells transfected with these mRNAs [8], [9], [10]. The elegant experiments by Pestova *et al*. [11] using reconstituted translation systems with all the purified components indicate that not all eIFs are necessary for EMCV translation *in vitro*. These investigators have observed that only a central domain of eIF4G was necessary for EMCV RNA translation, while eIF4E and eIF4B were dispensable [12]. The exclusion of eIF2 from these systems abolished protein synthesis directed by picornavirus mRNAs. The presence of IRES elements in mRNAs was also initially found in picornavirus mRNAs [6], [13]. The structure and the eIF requirements for the translation of the different IRES-containing picornavirus RNAs may vary among the different species investigated. Based on these differences, at present four classes of picornavirus IRESs can be considered [14], but all of them require eIF2 for efficient translation in cell free systems.

The function of eIF2 is to bind Met-tRNA_i_ and GTP to form the ternary complex Met-tRNA_i_-eIF2-GTP, which interacts with the P site on the 40S ribosomal subunit, establishing the interaction between the initiator AUG codon with the anticodon present in Met-tRNA_i_ [15], [16], [17]. Binding of the 60S ribosomal subunit to the pre-initiation complex promotes cleavage of GTP, displacing eIF2-GDP from the ribosome. The eIF2-GDP complex is recycled to eIF2-GTP by the activity of the recycling factor eIF2B. Factor eIF2 is composed of three subunits, known as α, β and γ [15], [16]. Subunit eIF2α is a 36 kDa protein that contains a serine residue at position 51 (Ser-51), which can be phosphorylated by four different cellular protein kinases. Nutrient deprivation or cellular stresses, such as heat-shock or viral infection, can activate some of these protein kinases [18], [19], [20]. GCN2 is activated by amino acid starvation, PKR phosphorylates eIF2 in response to double-stranded RNA, PERK is activated by protein misfolding at the endoplasmic reticulum and HRI phosphorylates eIF2 in the absence of HEME. Phosphorylation of eIF2α impairs the GDP-GTP recycling catalyzed by eIF2B. Therefore, the ternary complex Met-tRNA_i_ -eIF2-GTP is not generated and thus binding of this complex to the 40S ribosome is hampered. Even partial phosphorylation of eIF2 can lead to total abrogation of translation [21].

Study of eIF2 phosphorylation in picornavirus infected cells has yielded varying results. Some reports suggested that this factor remained unphosphorylated after poliovirus (PV) infection [22], [23], while other investigators found substantial eIF2 phosphorylation after PV infection, particularly at late times [24], [25]. Of interest, PKR becomes highly activated, yet it is hydrolyzed in PV-infected cells although this hydrolysis is not directly executed by any of the PV proteases (2A or 3C) [24], [26]. Mengovirus infection of mouse L-cells provokes a substantial activation of PKR, leading to eIF2 phosphorylation between 3–7 h after virus absorption [27]. The inactivation of eIF2 was coincident with the global inhibition of cellular and viral translation. Interferon treatment of culture cells stimulates, among others, PKR and the 2′-5′ A system blocking EMCV translation [28]. Direct evidence that activation of PKR alone suffices to block EMCV growth was provided by a cell line that stably synthesizes PKR [29]. All these findings pointed to the idea that active eIF2 was necessary to sustain picornavirus translation. The partial phosphorylation of eIF2 arising in picornavirus-infected cells as infection progresses might be partially responsible for the shut-down of cellular translation and the arrest of viral protein synthesis. Recent findings from our laboratory have provided evidence that Sindbis virus subgenomic mRNA exhibits a dual mechanism of translation. This mRNA follows a canonical mechanism when it is directly electroporated in cells or is translated in cell free systems, while it does not require eIF4G nor eIF2 for efficient translation in the infected cells [30]. A similar mechanism may be used by other viruses, including the Cricket paralysis virus [31]. In view of these findings, we reappraised the analysis for the participation of eIF2 during picornavirus RNA translation. Our present results indicate that EMCV protein synthesis does not require active eIF2 at late infection times, while this factor is necessary at early times, suggesting that EMCV mRNA translation can also follow a dual mechanism for the synthesis of viral proteins.

## Results

### Induction of eIF2α phosphorylation profoundly arrests cellular translation, while EMCV protein synthesis is resistant

**Figure 1:**
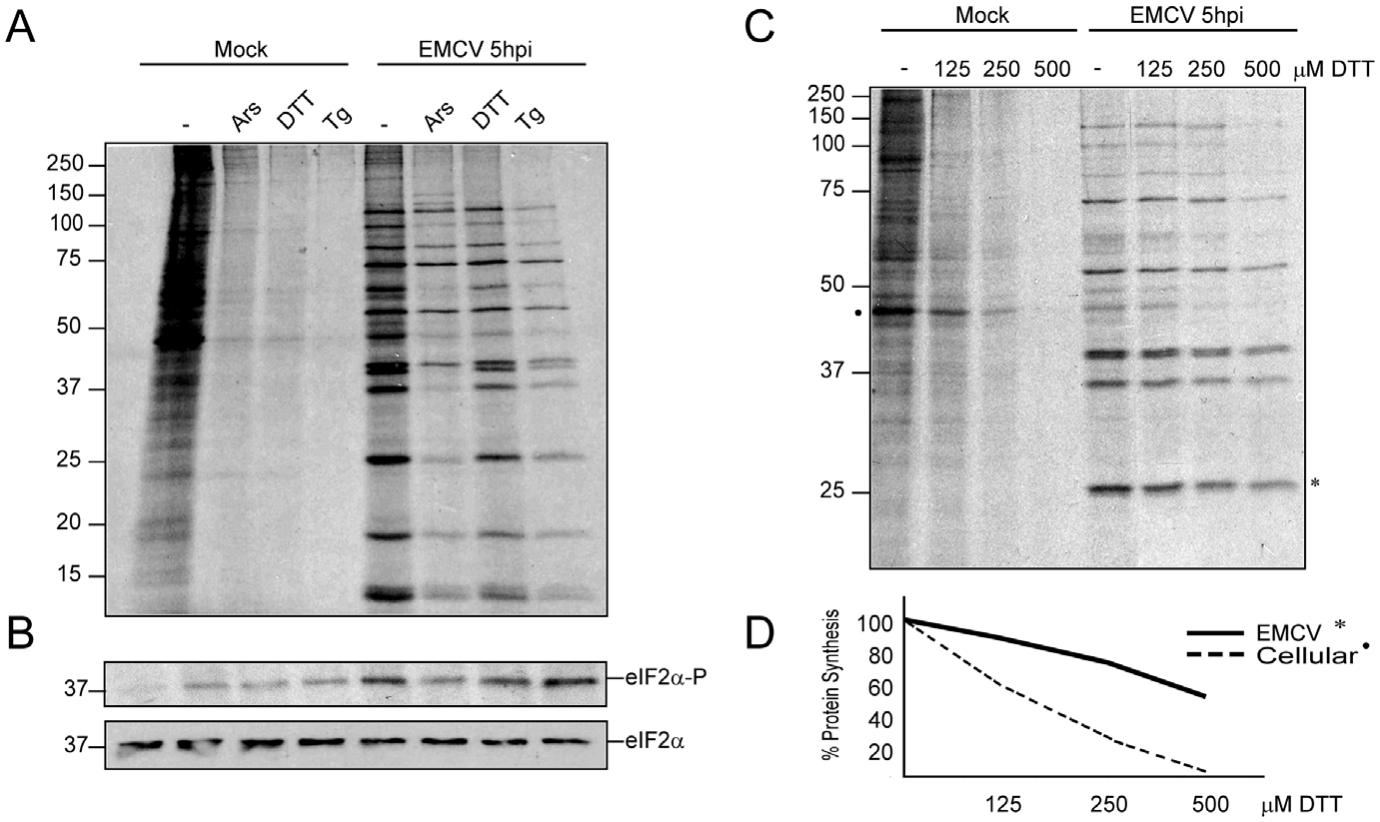
Effect of different inducers of eIF2α phosphorylation on cellular and EMCV translation. A) MEFs were mock- or EMCV-infected at 10 pfu/cell. Cells were subsequently pre-treated with 200 µM arsenite (Ars), 400 µM DTT, or 1 µM thapsigargin (Tg) for 15 min and then labelled for 45 min with [^35^S]Met-Cys in presence of the same compounds. Samples were submitted to SDS-PAGE, fluorography and autoradiography. B) Western blot analysis of eIF2α and phosphorylated eIF2α using the same samples as in panel A and antibodies anti-phospho-eIF2α (1∶1000 dilution) and anti-eIF2α (1∶1000 dilution). C) MEFs mock- or EMCV-infected at 10 pfu/cell were pre-treated for 15 min with 0, 125, 250 or 500 µM DTT and then labelled for 45 min with [^35^S]Met-Cys in presence of the same amounts of DTT. Samples were then collected and submitted to SDS-PAGE, fluorography and autoradiography. D) Cellular and viral protein synthesis examined by densitometric analysis of the autoradiograph shown in panel C. The protein bands analyzed are indicated by an asterisk.

Initially, we wished to test the effect of induction of eIF2α phosphorylation on EMCV translation in infected cells. Previous work has established that treatment of culture cells with compounds such as dithiothreitol (DTT), thapsigargin (Tg) or arsenite (Ars) causes phosphorylation of eIF2α leading to a profound arrest of cellular translation [32], [33]. Mouse embryo fibroblasts (MEFs) were infected or not with EMCV and at 4 hpi the test compounds were added to the medium and incubated for 1 h. Protein synthesis was estimated by addition of [^35^S]Met/Cys 15 min after the addition of the different compounds and incubated for 45 min. Protein synthesis was then analyzed by SDS PAGE followed by fluorography and phosphorylation of eIF2α was tested by Western blot (Figures 1A and B). Treatment with 400 µM DTT, 200 µM Ars and 1 µM Tg has no effect on the total amount of eIF2α, while the phosphorylated form of this factor clearly increases in the presence of any of these three compounds in control cells (Figure 1B). As a consequence, cellular protein synthesis strongly diminishes in the presence of these inhibitors (Figure 1A). By contrast, synthesis of EMCV proteins is almost unaffected by treatment with these agents, despite the fact that strong eIF2α phosphorylation is found in the infected cells. For instance, treatment of mock-infected cells with DTT induces 92% inhibition of cellular translation, as calculated by densitometric analysis, while EMCV protein synthesis only decreases by 24% (Figure 1A). Cellular translation was calculated by densitometry of the most prominent band that corresponds to actin, whereas viral translation was calculated by densitometry of all viral proteins. Notably, phosphorylation of eIF2α is clearly apparent in EMCV-infected cells at 5 hpi even in the absence of test compounds. This suggests that EMCV infection induces the phosphorylation of eIF2α.

It should be noted that Ars partially affects the proteolytic cleavage of the EMCV polyprotein, leading to the accumulation of viral precursors and the diminution of viral proteins of low MW. Therefore, we wished to test in more detail the action of DTT on cellular and viral translation. To this end, mock- or EMCV-infected cells were treated at 5 hpi with different DTT concentrations (125, 250 and 500 µM) and protein synthesis was measured from 5.15–6 hpi (Figure 1C). Increasing concentrations of DTT induce an almost total inhibition of cellular translation while EMCV protein synthesis is much less affected under these conditions (Figures 1C and 1D). These findings reveal that substantial EMCV protein synthesis occurs at late times of EMCV infection after induction of eIF2α phosphorylation by different compounds. To estimate the percentage of eIF2α phosphorylated by treatment of culture cells with DTT or Ars, isoelectric focusing was carried out. In untreated cells, most of eIF2α (95%) remains unphosphorylated, whereas in the presence of DTT or Ars almost all eIF2α (90–100%) becomes phosphorylated (Figure S1). These results agree well with our previous observations on the percentage of eIF2α phosphorylated in BHK cells infected with Sindbis virus and treated with Ars [30]. Therefore, this potent phosphorylation of eIF2α leads to the inhibition of cellular translation. The finding that Ars has little effect on late EMCV protein synhtesis suggests that this compound exhibits little toxicity on cellular processes that may influence mRNA translation, such as ATP or GTP synthesis. To further estimate the potential Ars toxicity on cellular protein synthesis, we employed the mouse cell line that expresses a form of eIF2α that cannot be phosphorylated. This cell line expresses an eIF2α bearing a point mutation at serine 51 (S51A). Addition of different Ars concentrations strongly inhibits cellular translation in control MEFs, whereas under these conditions Ars has almost no effect on protein synthesis in MEFs(S51A), demonstrating that the major effect of Ars on translation is mediated by the induction of eIF2α phosphorylation (Figure S2A). To further analyze the differential action of Ars in MEFs and MEFs(S51A), EMC-luc mRNA was transfected in these cells in presence or absence of Ars. Notably, luc synthesis was blocked in MEFs by about 85% in presence of Ars, whereas this compound had almost no effect in MEFs(S51A) (Figure S2B). However, we have found that, for unknown reasons, this variant cell line cannot be infected by several animal viruses tested, including EMCV and Sindbis virus.

### EMC-luc translation upon eIF2α phosphorylation in culture cells and in cell free systems

**Figure 2:**
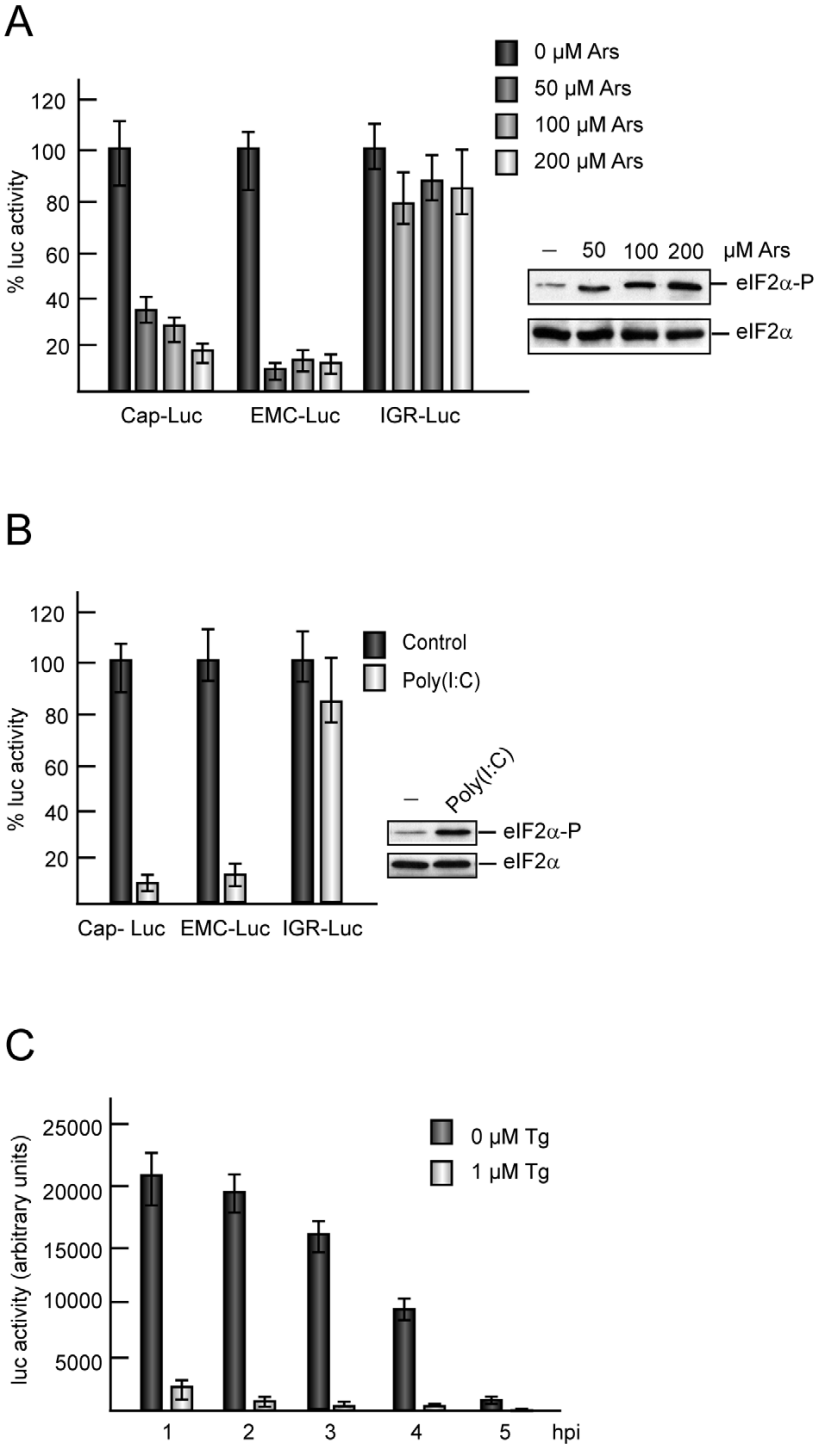
Translation of *in vitro* made mRNAs: Action of eIF2α phosphorylation. A) Cap-luc, EMC-luc, or CrPV IGR-luc mRNAs synthesized *in vitro* by T7 RNA polymerase were electroporated in BHK cells and seeded in DMEM (10% FCS). Different amounts of Ars (0, 50, 100 and 200 µM) were added and cells were incubated for 75 min before harvesting to analyze luc activity. The values shown are percentages of the value of their respective Ars untreated samples and are means ± SD of three independent experiments (left panel). The phosphorylated form of eIF2α and total eIF2α were determined in parallel by Western blot employing specific antibodies (right panel). B) Rabbit reticulocyte lysates were pre-treated or not with 0.5 µg/ml poly(I:C) for 30 minutes. Subsequently, 100 ng Cap-luc, EMCV-luc, or CrPV IGR-luc mRNAs were added and incubated for 1 h at 30°C. Luc synthesis was estimated by measuring luc activity. The values shown are percentages of the value of their respective poly(I:C) and are means ± SD of three independent experiments untreated samples (left panel). The phosphorylated form of eIF2α and total eIF2α were determined in parallel by Western blot (right panel). C) MEFs cells were infected with EMCV (10 pfu/cell) and next transfected with *in vitro* made EMC-luc mRNA at different times after infection. Cells were incubated for 75 min with the transcription mixture containing 5 µg EMC-luc mRNA for each L-24 well in presence or absence of 1 µM Tg and then collected to measure luc activity. Luc activity values are means ± SD of three independent experiments.

In Sindbis virus-infected cells, we have demonstrated that translation is coupled to transcription. Thus, late viral subgenomic mRNA exhibits a different requirement for eIFs when they are transcribed by the Sindbis virus replication machinery, as compared to their requirements when electroporated into culture cells [30], [34]. Overwhelming evidence obtained over the years in many laboratories has established that translation directed by EMCV RNA requires the participation of eIF2 [11], [35]. Therefore, our results described above indicating that eIF2 may not participate in the initiation of EMCV RNA translation were quite unexpected. In order to examine the requirement of eIF2 on translation driven by EMCV IRES, we used an EMC-luc mRNA synthesized by *in vitro* transcription, which contains the luc gene immediately behind the IRES sequence of EMCV. BHK cells were electroporated with EMC-luc and the action of Ars was tested. For comparative purposes cells were also electroporated with Cap-luc or CrPV IGR-luc mRNAs and then treated with different concentrations of Ars (0, 50, 100 and 200 µM) for 75 min. After that time luc activity was measured and the amount of phosphorylated eIF2α was analyzed (Figure 2A). At the highest dose of Ars, Cap-luc mRNA was inhibited by about 80%, while CrPV IGR-luc which is resistant to eIF2α phosphorylation was inhibited by only 20% (Figure 2A). Notably, luc synthesis directed by EMC-luc exhibited a high sensitivity to Ars, with 90% inhibition at 50 µM Ars. Analysis of eIF2α indicated that this factor was phosphorylated in Ars-treated cells (Figure 2A).

Next, *in vitro* translation of these different mRNAs was tested and the effect of poly(I:C) analyzed. For this purpose, rabbit reticulocyte lysates were programmed with EMC-luc, Cap-luc and CrPV IGR-luc mRNAs, in the absence or presence of the inhibitor. After incubation, luc activity was estimated. Poly(I:C) rendered an inhibition of EMC-luc translation of about 90%, similar to that found with Cap-luc, while CrPV IGR-luc was almost unaffected by this compound (Figure 2B). These results indicate that unphosphorylated eIF2α must be present in the cell or *in vitro* for efficient initiation of translation of EMC-luc. In addition, these findings contrast with those reported above (Figure 1), illustrating that late viral protein synthesis takes place when eIF2α is phosphorylated in EMCV-infected cells.

In EMCV-infected cells, preferential translation of viral mRNAs synthesized by viral transcription is observed [34]. Thus, EMC-luc mRNAs transfected in these cells at late times of infection are excluded from translation. Taking into account these considerations, we wanted to assay the effect of Tg on the translation of EMC-luc mRNA in EMCV-infected cells. To this end, EMCV-infected MEFs were transfected with EMC-luc mRNA at different hpi and the action of 1 µM Tg was tested (Figure 2C). Translation of exogenous EMC-luc mRNA decreases when it is transfected at late times of EMCV infection, in good agreement with our previous results [34]. Strikingly, Tg blocks EMC-luc mRNA translation at all hpi tested, pointing to a different behavior of EMCV RNA made from transcription or that transfected into cells, as regards to the requirement for active eIF2. Similar findings were obtained in BHK cells infected with EMCV and transfected with EMC-luc mRNA (Figure S3). It should be noted that EMCV translation becomes resistant to Tg inhibition as infection progresses. Thus, there is more inhibition of viral translation by Tg at 2–3 and 3–4 hpi than at 4–5 and 5–6 hpi. Once again EMC-luc transfected in these cells is excluded from translation, but Tg was able to strongly inhibit this residual luc synthesis. These observations suggest that in EMCV infected cells there is not a transacting viral factor that could confer eIF2-independence. Therefore, in the same infected cells, EMCV RNAs that are synthesized by the viral transcription machinery are more resistant to the phosphorylation of eIF2α than transfected EMC-luc mRNAs.

### Induction of eIF2α phosphorylation at the early stages of EMCV infection

**Figure 3:**
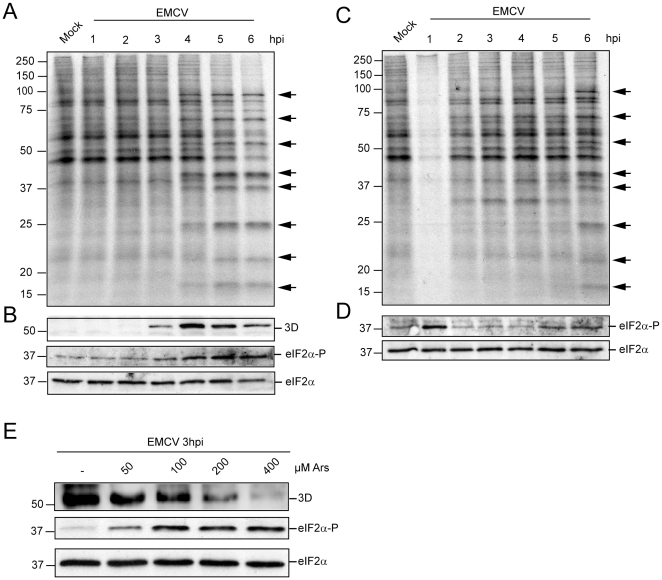
Treatment with Ars at early times of EMCV infection. A) MEFs were infected with EMCV (10 pfu/cell) and then protein synthesis was determined by labelling with [^35^S]Met-Cys every h from 1 to 6 hpi. B) Western blot analysis of the samples obtained in panel A using anti-3D, anti-phospho-eIF2α or anti-eIF2α antibodies. C) MEFs were infected with EMCV as in panel A and then treated with 200 µM Ars during 1 h (0–1 hpi). Next, Ars was washed and fresh medium was added. Protein synthesis was analyzed at the time of treatment with Ars and every h thereafter until 6 hpi. D) Western blot performed with anti-phospho-eIF2α or anti-eIF2α antibodies using the same samples as in panel C. E) MEFs cells were infected with EMCV (10 pfu/cell) and at 2 hpi treated with different amounts of Ars; one h later samples were harvested and the amount of polymerase 3D produced was determined by Western blot. The amount of eIF2α phosphorylated and total eIF2α was also determined. The arrows indicate viral proteins.

During the early stages of EMCV infection, genomic RNA is released to the cytoplasm for translation, whereas at late times of infection the viral mRNAs that participate in protein synthesis are produced by viral transcription. Our present results indicate that phosphorylation of eIF2α has little effect on viral protein synthesis in the late phase of EMCV infection. Therefore, we wanted to analyze the requirement for active eIF2 during the early stages of EMCV infection. To this end, MEFs were infected with EMCV and next treated or not with 200 µM Ars for 1 h. The cells were then washed and incubated with fresh medium and cell samples were collected at 1, 2, 3, 4, 5 and 6 hpi. EMCV proteins are evidenced by radioactive labelling at 4 hpi (Figure 3A, lane 4). In addition, the appearance of EMCV 3D polymerase can be evidenced by Western blot at 3 hpi (Figure 3B, lane 3). Strikingly, as the infection progresses, an increase in the phosphorylated form of eIF2α was observed (Figure 3B). When Ars is added at the beginning of infection, inhibition of cellular protein synthesis occurs (Figure 3C, lane 1) and this inhibition correlates with phosphorylation of eIF2α (Figure 3D, lane 1). After removal of Ars, the amount of phosphorylated eIF2α decreased to control levels, while cellular translation recovered. Significantly, viral protein synthesis is delayed, such that viral proteins start to be detected at 6 hpi (Figure 3C, lane 6). When eIF2α is phosphorylated at the beginning of infection, viral protein synthesis is delayed by about 2 h as compared to control cells. This finding suggests that for EMCV to begin translation, eIF2α needs to be dephosphorylated. To further analyze the effect of eIF2 phosphorylation on early translation of EMCV, cells were infected with EMCV, and at 2 hpi Ars was added at various concentrations (0, 50, 100, 200 and 400 µM). Then after 1 h of incubation, cells were harvested and samples were analyzed by Western blot using monoclonal anti-3D antibodies. Synthesis of EMCV 3D was strongly inhibited by the presence of Ars at these early times of infection, correlating with the phosphorylation of eIF2α (Figure 3E). In summary, translation of EMCV RNA is blocked at early times of infection if eIF2α is phosphorylated, while during the late phase of EMCV infection, viral protein synthesis can take place in the presence of phosphorylated eIF2α.

### Synthesis of EMCV proteins in cells with eIF2α depletion

**Figure 4:**
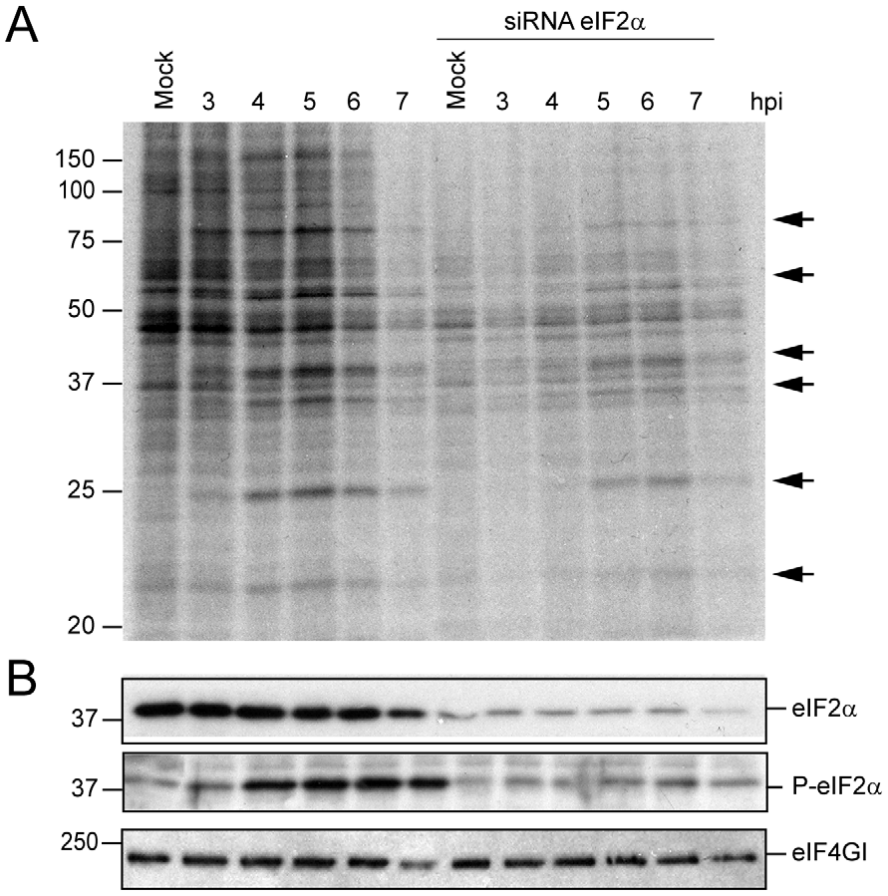
EMCV infection of Hela cells depleted or not of eIF2α. A) Hela cells transfected with a mixture of siRNAs targeting eIF2α mRNA or mock Hela cells were infected with EMCV (10 pfu/cell) at 36 h post-transfection. Next, protein synthesis was determined by [^35^S]Met-Cys labelling at the hpi indicated. Samples were analyzed by SDS-PAGE, fluorography and autoradiography. B) Western blot analysis of samples from panel A using anti-eIF2α or anti-phospho-eIF2α antibodies. As a control, the amount of eIF4GI in each sample was determined using specific antibodies against this factor. The arrows indicate viral proteins.

The use of siRNAs constitutes a useful tool to deplete eIFs in culture cells in order to examine their functioning during viral infection. A difficulty with this approach is that total depletion of the protein to be investigated is rarely achieved, but this approach may nevertheless indicate to what extent a given factor is involved in viral protein synthesis. Another potential problem is that some viral mRNAs may exhibit a dual mode of translation, requiring the factor early in the infection, but not at late times. In this case, a delay in viral protein synthesis may occur in those cells with partial depletion of the factor, while in strongly depleted cells, abrogation of viral translation and replication will occur. To assess the involvement of eIF2α in the translation of EMCV RNA, HeLa cells were depleted with siRNAs. To achieve this, cells were transfected with a mixture of four siRNAs designed to deplete eIF2α mRNA. At 36 h after siRNA transfection, HeLa cells were infected with EMCV. Samples were recovered at 3, 4, 5, 6 and 7 hpi and labeled proteins were analyzed (Figure 4A). Western blot analysis against eIF2α indicates that this factor is silenced by 90% (Figure 4B) and this depletion blocks cellular protein synthesis by 72% as estimated by densitometric analyses (Figure 4A). Notably, EMCV protein synthesis is delayed by about 1–2 h and strongly decreases as compared to undepleted cells infected with EMCV, although it can be clearly detected at late times of infection (6 and 7 hpi). The delay and decrease in EMCV translation in eIF2α-depleted cells are consistent with the idea that this factor participates in viral translation early during infection. To estimate the degree in which EMCV RNA synthesis is affected in eIF2α-depleted HeLa cells, [^3^H]uridine incorporation was estimated in presence of 5 µg/ml actinomycin D (Figure S4). A very strong inhibition was found in viral RNA replication consistent with the idea that early synthesis of viral proteins is necessary for genome replication. Despite this inhibition, once some viral RNA replication has taken place, translation of EMCV RNA at late times of infection shows little dependence on the presence of eIF2α (5–8 hpi).

### Subcellular localization of eIFs and ribosomes in EMCV-infected cells

**Figure 5:**
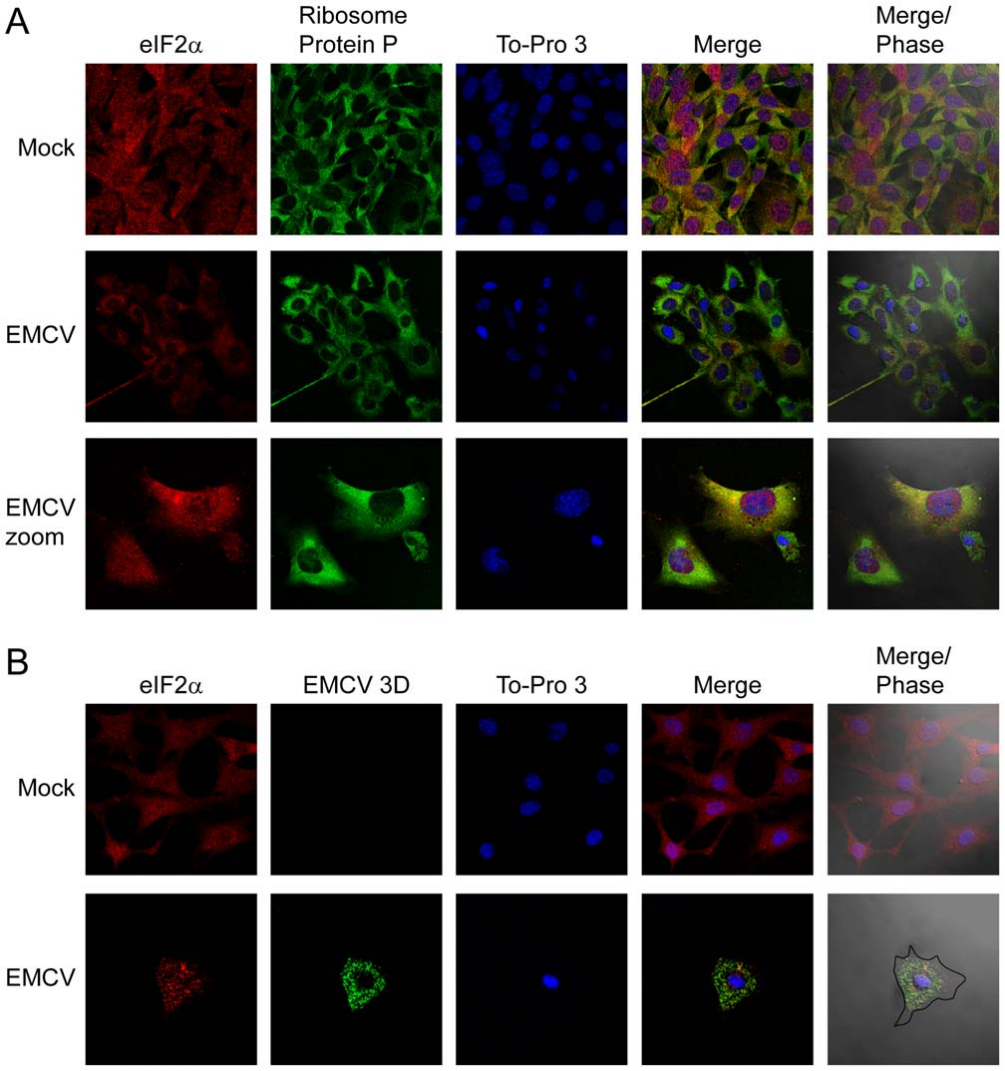
Subcellular localization of eIF2α, ribosomal protein P and EMCV 3D protein in Mock and EMCV infected cells. Hela cells were seeded on glass coverslips and mock infected or infected with EMCV (10 pfu/cell). At 5 hpi, cells were fixed and permeabilized. A) Ribosomal protein P and eIF2αwere detected by indirect immunofluorescence in mock- and EMCV-infected cells. ToPro 3 indicates the localization of the nucleus. B) Localization of eIF2α and EMCV 3D proteins. The cell outline was defined by differential interference contrast microscopy (Nomarski).

**Figure 6:**
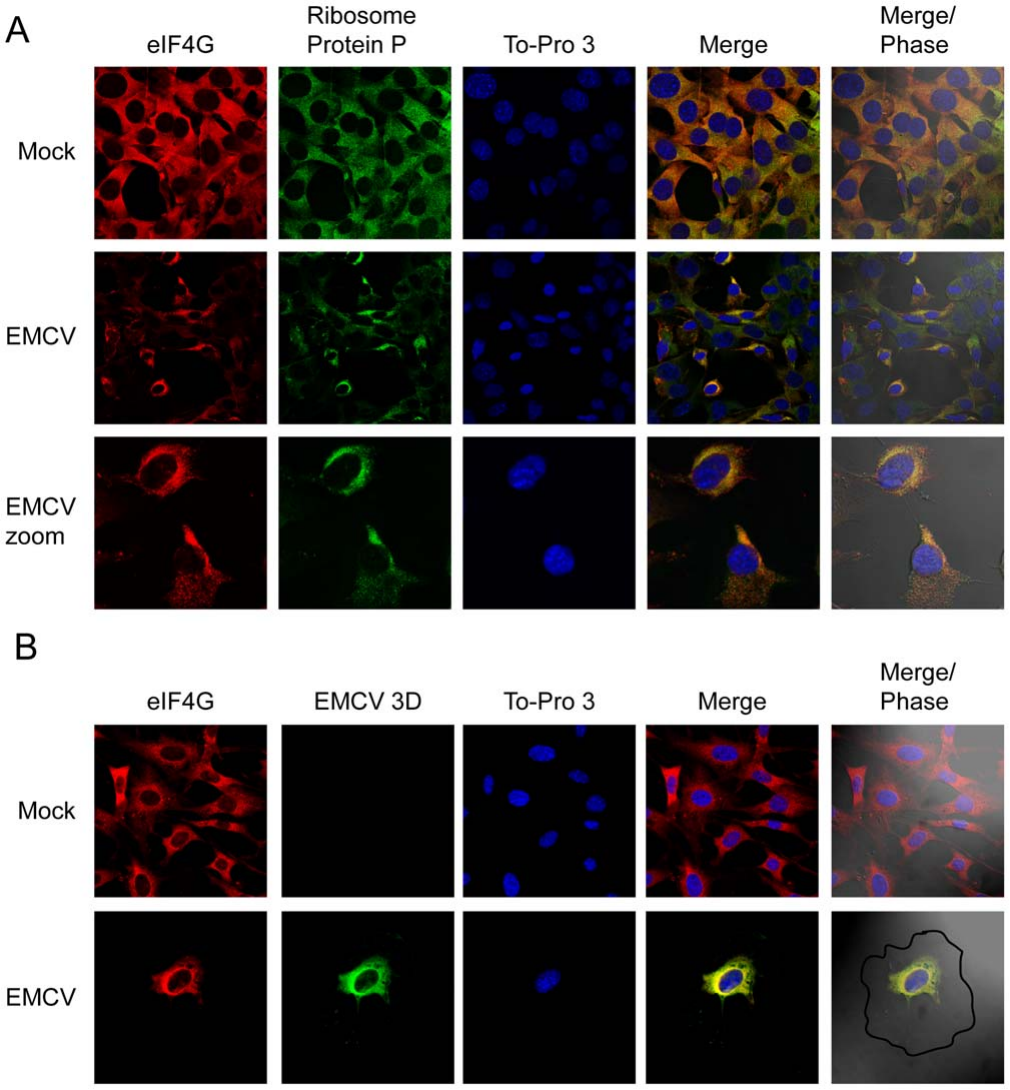
Subcellular localization of eIF4G, ribosomal protein P and EMCV 3D protein in infected cells. Hela cells were seeded on glass coverslips and mock infected or infected with EMCV (10 pfu/cell). At 5 hpi cells were fixed and permeabilized. A) Ribosomal protein P and eIF4G were detected by indirect immunofluorescence in mock- and EMCV-infected cells. ToPro 3 indicates the localization of the nucleus. B) Localization of eIF4G and EMCV 3D proteins. The cell outline was defined by differential interference contrast microscopy (Nomarski).

Another way to analyze the participation of eIFs in viral protein synthesis is to investigate their subcellular localization. Cytoplasmic animal viruses synthesize their proteins in a focal manner, particularly at late times of infection [30], [36], [37]. Ribosomes are present at those foci together with the eIFs that participate in translation, while those factors that are not involved in protein synthesis or viral replication are excluded from these foci. To investigate the subcellular localization of eIF2α after EMCV infection, MEFs were seeded on glass slides and infected or not with EMCV. At 5 hpi cells were fixed and incubated with the corresponding antibodies as indicated in Figures 5 and 6 prior to immunofluorescence analysis. In mock infected cells, eIF2α colocalizes with ribosomal protein P in the cytoplasm (Figure 5A). By contrast, those two proteins do not colocalize in EMCV infected cells (Figure 5A). EMCV 3D polymerase is clearly observed in the cytoplasm, indicating the viral replicative sites (Figure 5B). Both the cytoplasmic sites for viral translation and RNA replication are located in a perinuclear region and overlap, consistent with the idea that transcription and translation are coupled processes [34]. Notably, EMCV 3D does not colocalize with eIF2α. Using the ImageJ program with the Just Another Co-localization Plugin (JaCoP), the colocalization rate was calculated [38]. For eIF2α and ribosomal protein P the Pearson's Coefficient was 0.96 on the 0 to 1 scale (0–0.5 indicates no colocalization and 0.5–1, colocalization), indicating almost total colocalization between eIF2α and ribosomes in mock-infected cells, while in EMCV infected cells this coefficient was 0.28, suggesting that there was no colocalization for eIF2α and ribosomes. In the case of eIF2α and EMCV 3D protein, the Pearson Coefficient was 0.32, which further suggests that there is no colocalization between those two proteins. Therefore, eIF2 is excluded from viral replicative foci.

To compare the above results with other factors that are involved in EMCV translation, eIF4G localization was investigated. This initiation factor is present in the cytoplasm around the nucleus colocalizing completely with ribosomal protein P in both mock and EMCV infected cells (Figure 6A); the Pearson Coefficient for eIF4G and ribosomal protein P was 0.89, and 0.92, respectively. In addition, eIF4GI also colocalizes with EMCV 3D protein (Figure 6B) (Pearson's coefficient 0.9) suggesting that eIF4G participates in EMCV translation. Therefore, the results obtained on the subcellular localization of eIF2α further support the notion that eIF2, contrary to eIF4GI, is not involved in the initiation of EMCV protein synthesis.

### Requirement of active eIF2 for RNA translation with other picornaviruses

**Figure 7:**
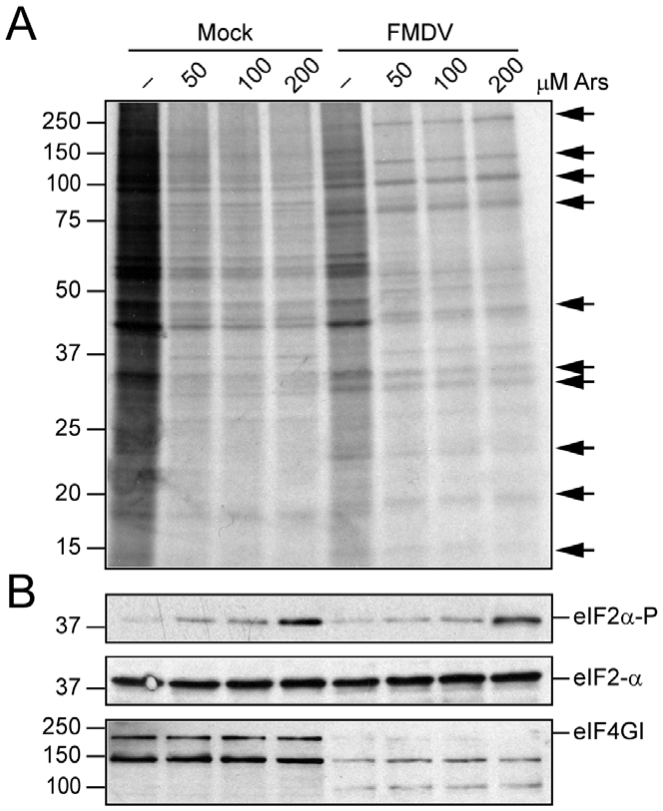
Action of Ars on FMDV infection. A) BHK cells were mock infected or infected with FMDV (10 pfu/cell). At 3 hpicultures were treated with different amounts of Ars (0, 50, 100 and 200 µM) for 15 min and next labeled by [^35^S]Met-Cys labelling in presence of the same concentrations of Ars from 3.15–4 hpi. Samples were collected and analyzed by SDS-PAGE, fluorography and autoradiography. B) The phosphorylated form of eIF2α and total eIF2α were determined in parallel by Western blot with specific antibodies. The cleavage of eIF4GI was also analyzed by Western blot using specific antibodies against this factor. The arrows indicate viral proteins.

After demonstrating that EMCV RNA exhibits a dual mode for translation—i.e. this RNA requires the presence of active eIF2 in infected cells early during infection, but not at late times—we wished to examine the involvement of eIF2 in RNA translation of other picornaviruses. For this purpose, BHK cells were infected with FMDV, a member of the Aphtovirus genus, and at 3 hpi Ars (50, 100 and 200 µM) was added to the medium and incubated for 1 h. Protein synthesis was estimated by incubation with [^35^S]Met/Cys during 45 min, from 3.15–4 hpi in the presence or absence of Ars. Cells were then collected and the synthesized proteins analyzed (Figure 7A). Phosphorylation of eIF2α and cleavage of eIF4GI were also analyzed (Figure 7B). As expected, addition of Ars strongly induced eIF2α phosphorylation. No inhibition of FMDV protein synthesis was observed by Ars under all the concentrations tested. By contrast, cellular translation was almost totally blocked at 100 µM Ars. These findings clearly indicate that FMDV RNA translation takes place in the presence of phosphorylated eIF2α during the late phase of infection.

**Figure 8:**
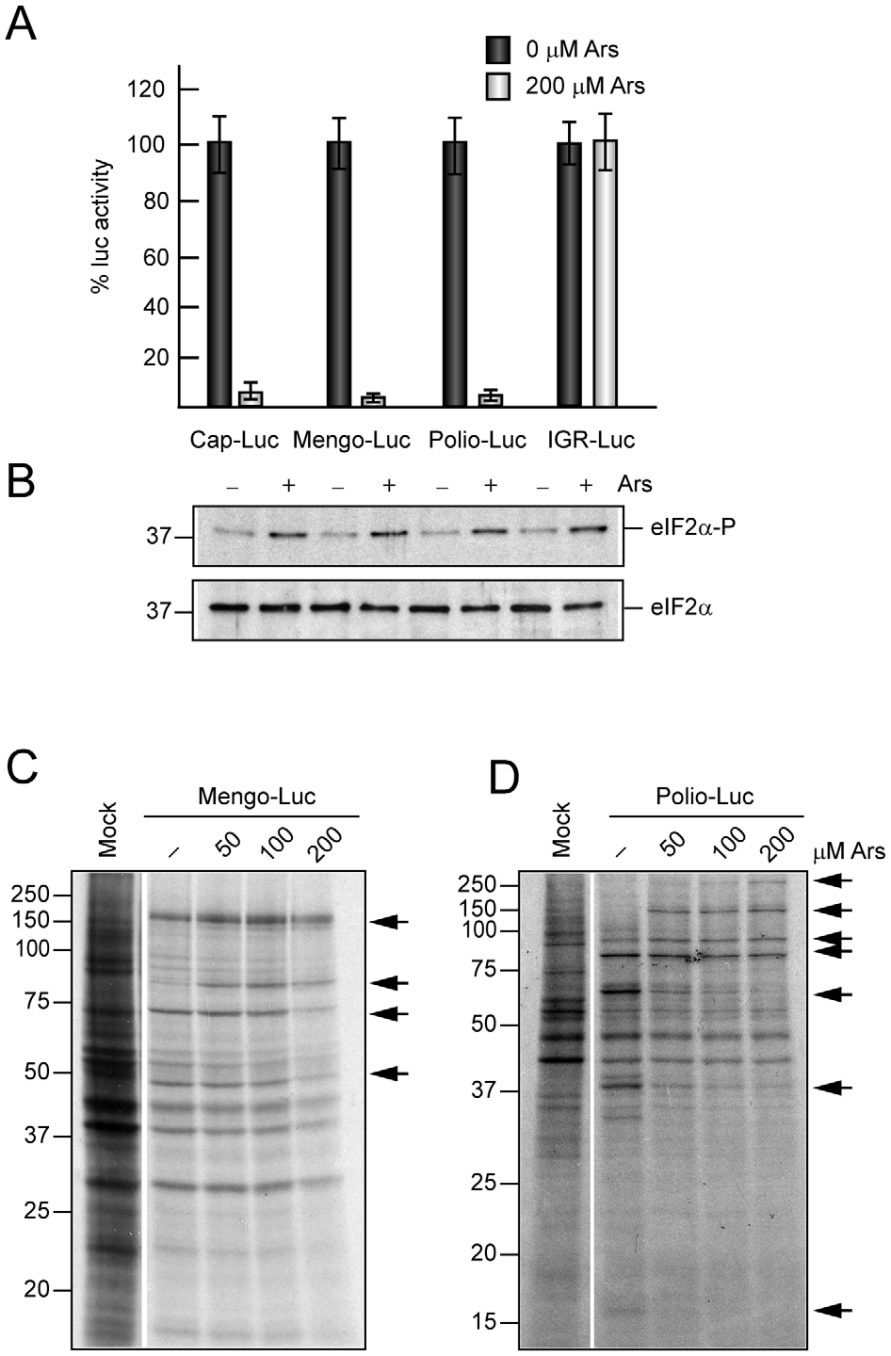
Translation of Mengovirus and PV replicons. Effect of Ars on early and late viral protein synthesis. A) BHK cells were electroporated with Mengo-luc, Polio-luc replicons, Cap-luc or CrPV IGR-luc mRNAs; all these RNAs were synthesized by *in vitro* transcription. Electroporated cells were seeded in DMEM (10% FCS) in presence or absence of 200 µM Ars. 75 min later cells were collected and lysed to measure luc activity. The values shown are percentages of the value of their respective Ars untreated samples and are means ± SD of three independent experiments. B) The phosphorylated form of eIF2α and total eIF2α were determined in parallel by Western blot with specific antibodies. C and D) BHK cells were electroporated with Mengo-luc replicon (C) or polio-luc replicon (D) and at 5 h post-electroporation protein synthesis was determined by [^35^S]Met-Cys labelling in presence of different concentrations of Ars (0, 50, 100 and 200 µM). The arrows indicate viral proteins.

To further analyze the effect of inducers of eIF2α phosphorylation on early and late protein synthesis in picornavirus-infected cells, two replicons, one from mengovirus and another from PV, were analyzed. Both replicons contain the luc gene replacing the coding region for viral structural proteins. Mengovirus is closely related to EMCV and both belong to the Cardiovirus genus, while PV is the prototype member of the Enterovirus genus. These replicons have the advantage that early translation can be assayed by estimating luc synthesis, whereas the synthesis of late proteins can be examined by radioactive labelling when the shut-off of host translation has occurred. After electroporation of these replicons into BHK cells, 200 µM Ars were added to the culture medium and luc activity was measured at 75 min. As controls Cap-luc and CrPV IGR-luc were used. Remarkably, luc synthesis from each replicon, as well as from Cap-luc mRNA, was drastically inhibited by Ars, whereas luc synthesis directed by CrPV-luc was unaffected in the presence of Ars (Figure 8A). The effect of this compound on late viral translation was assayed using different Ars concentrations (50, 100 and 200 µM). At late times of replication (5 h post-electroporation), Ars has little effect on Mengovirus protein synthesis (Figure 8C). Thus, only 30% inhibition was found in the presence of 200 µM Ars, whereas cellular translation usually diminished by over 90% under these conditions. The PV replicon exhibited a similar behavior to the Mengovirus one as regards the inhibitory action of Ars (Figure 8D). Altogether, these results provide strong evidence that synthesis of picornavirus proteins does not require eIF2α during the late phase of infection.

## Discussion

Participation of eIF2 in the formation of the ternary complex Met-tRNA_i_–eIF2-GTP is a crucial event in the initiation of translation of most mRNAs whether of cellular or viral origin [16], [17]. However, mRNA translation of some viruses, such as HCV or CrPV, does not require this factor [39], [40], [41], [42]. Our present observations indicate that picornavirus RNA translation takes place when eIF2α is phosphorylated, revealing that this factor is not necessary to translate this RNA at late times of infection. If so, the functioning of IRES elements from HCV, CrPV IGR, and picornaviruses reflects more similarities than previously suspected. Moreover, some cellular mRNAs bearing IRES elements can also be translated when eIF2α becomes phosphorylated [43], [44].

### Dual mechanism for EMCV translation

The concept that mRNA structure determines the mechanism by which translation takes place has not been supported by our recent findings demonstrating that a viral mRNA such as Sindbis virus subgenomic (SV sg-mRNA) exhibits a dual mode for initiation of translation [30]. This mRNA requires active eIF2 and intact eIF4F complex when it is translated in cell free systems or when electroporated in culture cells. However, SV sg-mRNA efficiently directs translation in the presence of phosphorylated eIF2α or upon eIF4G cleavage in virus-infected cells [30], [45]. Consistent with these findings, our present results support the notion that picornavirus RNA also exhibits a dual mode for its translation. Thus, EMCV RNA is efficiently translated at late times of infection when eIF2α has been phosphorylated. By contrast, as shown in this and other studies, *in vitro* protein synthesis driven by EMCV IRES is profoundly blocked upon phosphorylation of eIF2α [41], [46]. A similar situation is found when this IRES containing RNA is transfected in cells or at early times of EMCV infection. Our conclusion is that EMCV RNA can be translated following a canonical mechanism as regards to the use of eIF2, early during infection. As infection progresses, the cellular environment is modified such that this RNA can now direct translation in the absence of active eIF2. Therefore, EMCV RNA has a dual mode for translation, despite the fact that this RNA possesses the same structure at early and late times of infection. If true, the mechanism by which picornavirus RNA is translated would depend on two parameters: 1) the structure of this mRNA and 2) the environment in which translation is examined. In addition, our present findings provide an explanation for the partial resistance of EMCV in cells that express PKR [29].

The dual mode of translation for viral mRNAs occurs not only with SV sg- mRNA and picornavirus RNAs, but also with CrPV mRNA [31]. We have speculated that the presence of viral proteins is responsible for the switch between these two modes to initiate translation [30]. In this regard, Hantavirus N protein is able to replace the eIF4F complex, thus the mechanism of viral translation in this instance is due to N protein [47]. Also, PV 2A^pro^ can rescue the translatability of SV sg-mRNAs bearing a picornavirus IRES [48].

### Picornavirus translation in the presence of phosphorylated eIF2

It is puzzling to envisage how EMCV RNA initiation might occur in the absence of eIF2. Several possibilities exist: one is that a cellular protein or factor can act as a substitute for eIF2. This may be the case for HCV RNA translation, where eIF5B acts as a substitute for eIF2 [39]. It has also been proposed that eIF2A could act as a substitute for eIF2 in infections with Sindbis virus [49]. These data have been questioned recently as it has been suggested that other cellular proteins such as ligatin or MCT-1 and DENR can replace eIF2 during the initiation of HCV protein synthesis or during the translation of SV sg-mRNA [50]. However, the authors of that study did not find that ligatin can replace eIF2 for the initiation on EMCV RNA. In fact, ligatin has been identified as eIF2D [51]. This factor can replace eIF2 for the translation of some cellular mRNAs. Binding of aminoacyl-tRNA to the ribosomal P-site is promoted by eIF2D in a GTP-independent fashion [51]. In principle, it should be possible that the function of eIF2 was replaced by eIF2A or eIF2D in picornavirus infected cells. Another possibility is that the IRES itself directly binds to the 40S, or even to the 80S ribosome, at the P site late during infection, directly triggering the elongation phase. If so, the activity of picornavirus IRESs may be more similar to CrPV IGR IRES than previously thought [40], [52]. Therefore, to know exactly which mechanism is acting during initiation with the different IRES-containing mRNAs thus far identified in virus species, the mechanism of translation has to be examined in virus-infected cells. The results obtained in cell free systems or even in culture cells transfected with these mRNAs may be misleading and cannot be extrapolated to the physiological situation. As illustrated in the present work, the mechanism for initiation of translation on EMCV RNA requires active eIF2 *in vitro*, this being the mode of translation closer to the canonical mechanism than that observed in the infected cells during the bulk of viral translation.

### A variety of mechanisms to initiate translation on viral mRNAs

Two major mechanisms for the initiation of translation are known in eukaryotic cells: m7G cap-dependent or m7G cap-independent [16], [17]. This division is mainly based on whether or not a m7G cap structure is present at the 5′ end of mRNAs and/or the requirement for eIF4E during mRNA translation. However, this simplistic classification may lead to some confusion because there are capped mRNAs that do not require eIF4E. In some instances, such as Adenoviruses, Influenza virus or Hantavirus, a viral protein recognizes the m7G cap structure of viral mRNA and replaces eIF4E or even eIF4F [47], [53], [54]. Thus, translation depends on the presence of a m7G cap structure, but eIF4E is dispensable. This is also the case of SV sg-mRNA, which does not require intact eIF4G but still needs the m7G cap structure at the 5′-end of this mRNA [45], [48]. When defining the mechanism of initiation it seems more adequate to refer to the eIFs that are involved in translation [55]. According to whether eIF2 is required for translation, one of two different mechanisms of initiation is defined. One is the canonical mechanism that uses the ternary complex Met-tRNA_i_-eIF2-GTP while the other does not require this factor. In this last case a variety of mechanisms can be operative depending on the type of mRNA examined and the conditions analyzed. The situation is that depending on the cellular or viral mRNA considered and the type of assay employed (*in vitro*, intact cells, stress situations, viral infections, etc.) the requirements for eIFs can vary. This picture may be slightly more complicated if we bear in mind that a given mRNA can exhibit different mechanisms of initiation, reflecting the plasticity of some RNAs in accommodating stress situations.

## Materials and Methods

### Cell Cultures and Viruses

The cell lines used in this work were: HeLa, BHK-21 and mouse embryo fibroblasts (MEFs). The mouse cell line MEFs(S51A) that contains an unphosphorylatable form of eIF2α was kindly provided by D. Ron and R.J. Kaufman (Department of Biological Chemistry, MI, USA). Cells were grown at 37°C in Dulbecco's Modified Eagle's Medium (DMEM) supplemented with 5% fetal calf serum (FCS) (HeLa and BHK) or 10% FCS (MEFs) and nonessential amino acids. Infection with EMCV or with foot-and-mouth disease virus (FMDV) was carried out at a multiplicity of 10 pfu/cell.

### Plasmids

The constructs pKs luc and pTM1-luc have been already described [48]. These plasmids were used as DNA template to obtain Cap-luc and EMC-luc mRNA by *in vitro* transcription using the T7 RNA polymerase. Plasmid T7 Rluc ΔEMCV IGR-Fluc [56] was employed to obtain CrPV IGR-luc mRNA. The constructs encoding the PV replicon pRluc31 [57] and the mengovirus replicon RZ-pMluz [58] have been already described.

### Protein metabolic labeling and Western blot analysis

Protein synthesis was analyzed by replacing DMEM growth media with 0.2 ml methionine–cysteine free DMEM supplemented with 2 µl EasyTag™ EXPRESS ^35^S Protein Labeling mix, [^35^S]Met-Cys (11 mCi/ml, 37.0 Tbq/mmol; Perkin Elmer) per well of an L-24 plate. Cultures were pre-treated with the amounts indicated in each case of dithiothreitol (DTT), thapsigargin (Tg) or arsenite (Ars) for 15 min, before labeling for 45 min in the presence of the tested compounds. The cells were then collected in the appropriate gel loading buffer (62.5 mM Tris–HCl, pH 6.8, 2% SDS, 0.1 M DTT, 17% glycerol, and 0.024% bromophenol blue) and analyzed by electrophoresis in SDS-polyacrylamide gels (SDS-PAGE), followed by fluorography and autoradiography. Specific rabbit polyclonal antibodies raised against phospho-eIF2α (Ser 51) (Cell Signaling Technology) or total eIF2α (Santa Cruz Biotechnology) were used in Western blot analysis at 1∶1000 dilution antisera. Mouse monoclonal antibodies raised against EMCV 3D protein (a generous gift from A. Palmenberg, University of Wisconsin, Madison, USA) were used at 1∶1000 dilution. Rabbit polyclonal antibodies raised against peptides derived from the N- and C-terminal regions of human eIF4GI were also used at a 1∶1000 dilution [59]. Anti-rabbit and anti-mouse immunoglobulin G antibodies coupled to peroxidase (Promega) were used at a 1∶5000 dilution.

### *In vitro* transcription and transfection

Plasmids were used as templates for *in vitro* RNA transcription with T7 RNA polymerase (Promega). To obtain Cap-luc mRNA, an m^7^G(5′)ppp(5′)G cap analog was added to the transcription mixture. For transfection, subconfluent BHK cells were harvested, washed with ice-cold phosphate-buffered saline (PBS), and resuspended at a density of approximately 2.5×10^6^ cells/ml in the same buffer. Subsequently, 20 µg of *in vitro* transcribed RNA were added to 0.4 ml cell suspension and the mixture was transferred to a 2-mm cuvette. Electroporation was carried out at room temperature by generating two consecutive 1.5-kV, 25-mF pulses with a Gene Pulser apparatus (Bio-Rad), as previously described [48].

### *In vitro* translation

*In vitro* translation was carried out in rabbit reticulocyte lysates. To induce phosphorylation of eIF2α, extracts were treated with 0.5 µg/ml poly(I:C) (Pharmacia Biotech) for 30 min. Subsequently, 100 ng of different mRNAs were added and incubated for 1 h at 30°C. Protein synthesis was estimated by measuring luc activity.

### Transfection of HeLa cells

To transfect interference RNAs (siRNAs), HeLa cells were grown in 24-well plates with antibiotic- and antimycotic free DMEM supplemented with 5% FCS to 60–70% confluence. To make up the transfection mixture, 2 µl of Lipofectamine 2000 (Invitrogen) were added to 50 µl of Opti-MEM I Reduced Serum Medium (Opti-MEM I) (Invitrogen) and then incubated for 5 min at room temperature. Simultaneously, the siRNA mixture was prepared with 100 pmol of a mixture of four siRNAs targeting eIF2α mRNA (Dharmacon; Thermo Scientific) in 50 µl of the Opti-MEM I for each L-24 well and then incubated at room temperature for 5 min. The final mixture was subsequently prepared with 50 µl of Lipofectamine suspension and 50 µl of the siRNA mixture by incubation for 30 minutes at room temperature. To transfect HeLa cells with siRNAs, cell medium was removed and 100 µl of Opti-MEM I followed by 100 µl of the transfection/siRNAs mixture obtained were added to each well. Cells were then incubated at 37°C for 4 h. After incubation, the transfection medium was removed and the cultures continued in fresh medium. At 36 h post-transfection HeLa cells were infected with EMCV (10 pfu/cell) to determine viral protein synthesis.

### Luciferase activity measurement

HeLa cells were harvested with buffer containing 25 mM glycylglycine (pH 7.8), 0.5% of Triton X-100 and 1 mM dithiothreitol. Luciferase (luc) activity was measured using Moonlight 2000 apparatus (Analytical Luminescence Laboratory) using the Luciferase Assay System (Promega).

### Immunofluorescence analysis

HeLa cells were seeded on glass cover slips prior to infection with EMCV (10 pfu/cell). At 5 hpi, cells were fixed in 4% PFA for 15 min, washed twice with PBS, and then permeabilized for 10 min with 0.2% Triton X-100 in PBS. Subsequent antibody incubations were carried out for 2 h with specified primary antisera and corresponding fluorescence-conjugated secondary antibody at room temperature. Cover slips were then mounted in ProLong Gold anti-fade reagent (Invitrogen) and examined with a Zeiss LSM510 Inverted confocal laser-scanning microscope (Bio-Rad/Zeiss) with Plan-Apochromat 63X/1.4 oil objective. Mouse monoclonal antibodies raised against eukaryotic ribosomal P protein [60], or EMCV 3D protein (a gift from A. Palmenberg, University of Wisconsin, Madison, USA) were used for immunofluorescence at 1∶10 and 1∶200 dilutions, respectively. Rabbit polyclonal antibodies raised against eIF4GI or eIF2α were used at 1∶100 dilution. To-pro3 (Invitrogen) was employed at 1∶500 dilution.

## Supporting Information

Figure S1 **Analysis of phosphorylated and unphosphorylated eIF2α in culture cells. Effect of inhibitors.** HeLa cells were untreated or treated for 60 min with 200 µM Ars or 400 μM DTT. Afterwards cell monolayers were collected and proteins were separated by isoelectric focusing and transferred to a nitrocellulose membrane as described before [30]. The phosphorylated and unphosphorylated forms of eIF2α were detected by anti-eIF2α rabbit polyclonal antibodies and quantified by densitometric scanning of the corresponding bands.
(TIF)

Figure S2 **Effects of Ars on translation in MEFs.** A) Protein synthesis was analyzed in MEFs or MEFs(S51A) treated with different concentrations of Ars as indicated in the Figure. Culture cells were pretreated for 15 min with Ars in DMEM without methionine and cysteine. Then, 15 µCi of [^35^S]Met-Cys for each L-24 well were added and incubation was continued for 1 h. Cells were collected in sample buffer and proteins synthesized during this time were analyzed by SDS-PAGE, fluorography and autoradiography as described in Materials and Methods. B) Luc synthesis in MEFs or MEFs(S51A) transfected with EMC-luc mRNA in the presence of different concentrations of Ars. Culture cells were transfected with 5 μg of EMC-luc mRNA per well of an L-24 plate in the presence of 0, 200 or 400 μM Ars. 75 min later cell monlayers were collected and lysed to measure luc activity. The percentage to the values of the respective samples untreated with Ars is represented. Luc activity values are means ± SD of three independent experiments.
(TIF)

Figure S3 **Translation of EMC-luc mRNA transfected in EMCV-infected cells.** **A**) BHK cells were infected with EMCV (10 pfu/cell) and next transfected with EMC-luc mRNA at different times after infection. The cells were incubated for 75 min with the transcription mixture containing 5 μg EMC-luc mRNA per well of an L-24 plate in presence or absence of 1 μM Tg and then collected to measure luc activity. Luc activity values are means ± SD of three measures of the same experiment. B) Protein synthesis was analyzed in parallel. In this case the cultures were treated or not with 1 μM Tg for 15 min before adding 15 µCi of [35S]-Met, Cys per well of an L-24 plate, and continue the incubation for 1 h. The arrows indicate viral proteins.
(TIF)

Figure S4 **EMCV RNA synthesis in eIF2-depleted HeLa cells.** Hela cells transfected with a mixture of siRNAs targeting eIF2αmRNA or mock Hela cells were infected with EMCV (10 pfu/cell) at 36 h post-transfection. Viral RNA was subsequently labeled with [^3^H]uridine (20 µCi/ml, final concentration) in the presence of 5 µg/ml actinomycin D. At the indicated hpi [^3^H]uridine incorporated was quantified in a liquid scintillation spectrometer as described before [48]. Cpm values are means ± SD of three measures of the same experiment.
(TIF)
