# Supplementary figures and images for: Translation of Viral mRNA without Active eIF2: The Case of Picornaviruses
Source: PLoS One. 2011 Jul 14;6(7):e22230. doi: 10.1371/journal.pone.0022230 (PMC3136507; doi:10.1371/journal.pone.0022230)

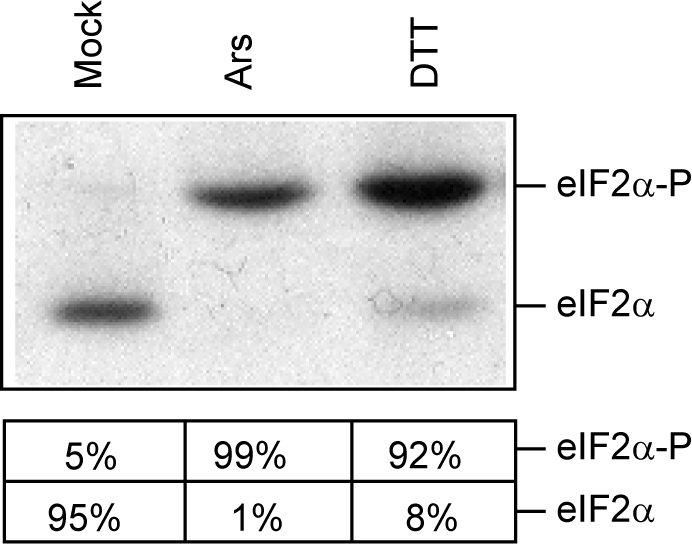

Supplement: Figure S1 — Analysis of phosphorylated and unphosphorylated eIF2α in culture cells. Effect of inhibitors. HeLa cells were untreated or treated for 60 min with 200 µM Ars or 400 μM DTT. Afterwards cell monolayers were collected and proteins were separated by isoelectric focusing and transferred to a nitrocellulose membrane as described before [30]. The phosphorylated and unphosphorylated forms of eIF2α were detected by anti-eIF2α rabbit polyclonal antibodies and quantified by densitometric scanning of the corresponding bands. (TIF) [file pone.0022230.s001.tif]

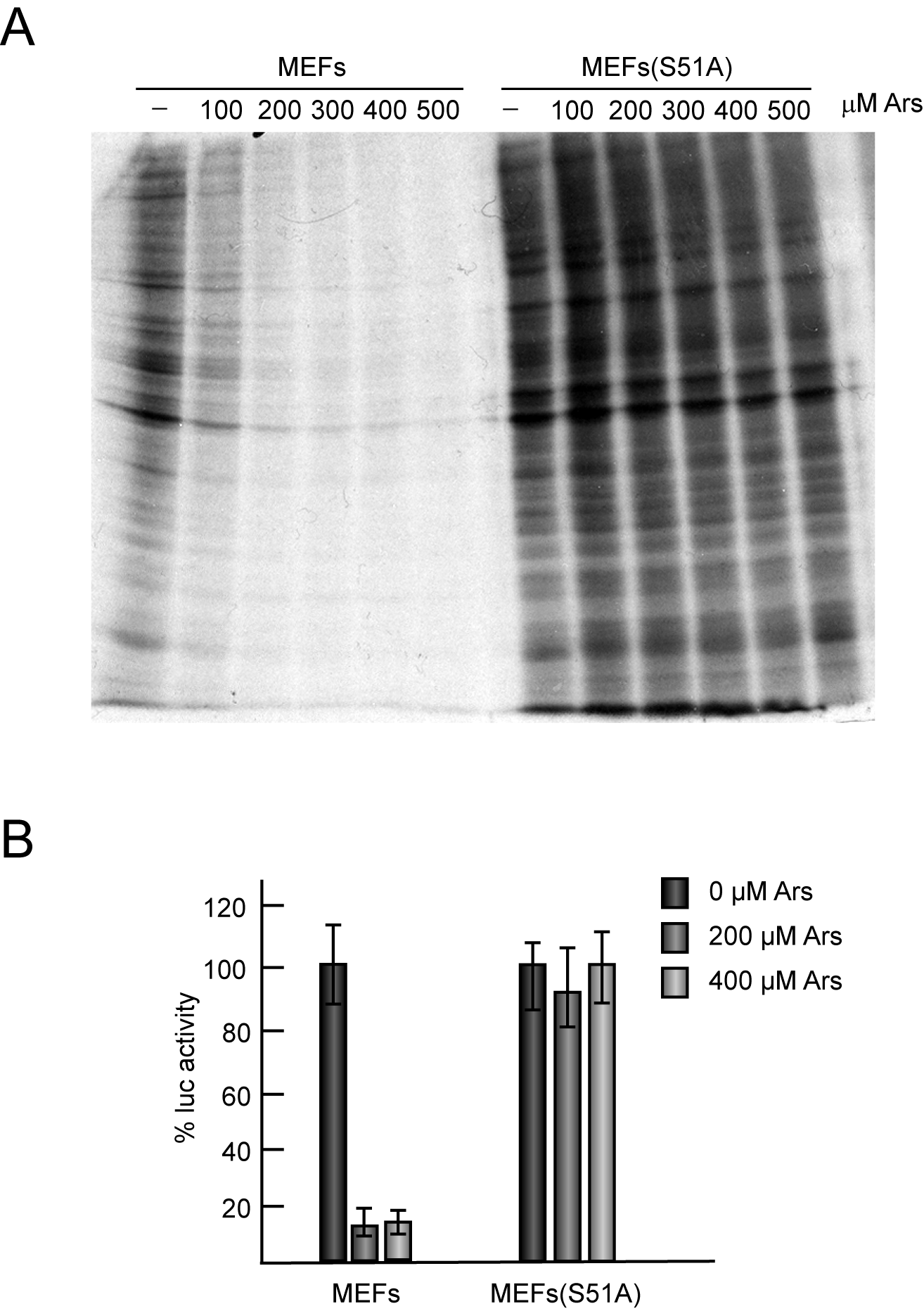

Supplement: Figure S2 — Effects of Ars on translation in MEFs. A) Protein synthesis was analyzed in MEFs or MEFs(S51A) treated with different concentrations of Ars as indicated in the Figure. Culture cells were pretreated for 15 min with Ars in DMEM without methionine and cysteine. Then, 15 µCi of [35S]Met-Cys for each L-24 well were added and incubation was continued for 1 h. Cells were collected in sample buffer and proteins synthesized during this time were analyzed by SDS-PAGE, fluorography and autoradiography as described in Materials and Methods. B) Luc synthesis in MEFs or MEFs(S51A) transfected with EMC-luc mRNA in the presence of different concentrations of Ars. Culture cells were transfected with 5 μg of EMC-luc mRNA per well of an L-24 plate in the presence of 0, 200 or 400 μM Ars. 75 min later cell monlayers were collected and lysed to measure luc activity. The percentage to the values of the respective samples untreated with Ars is represented. Luc activity values are means ± SD of three independent experiments. (TIF) [file pone.0022230.s002.tif]

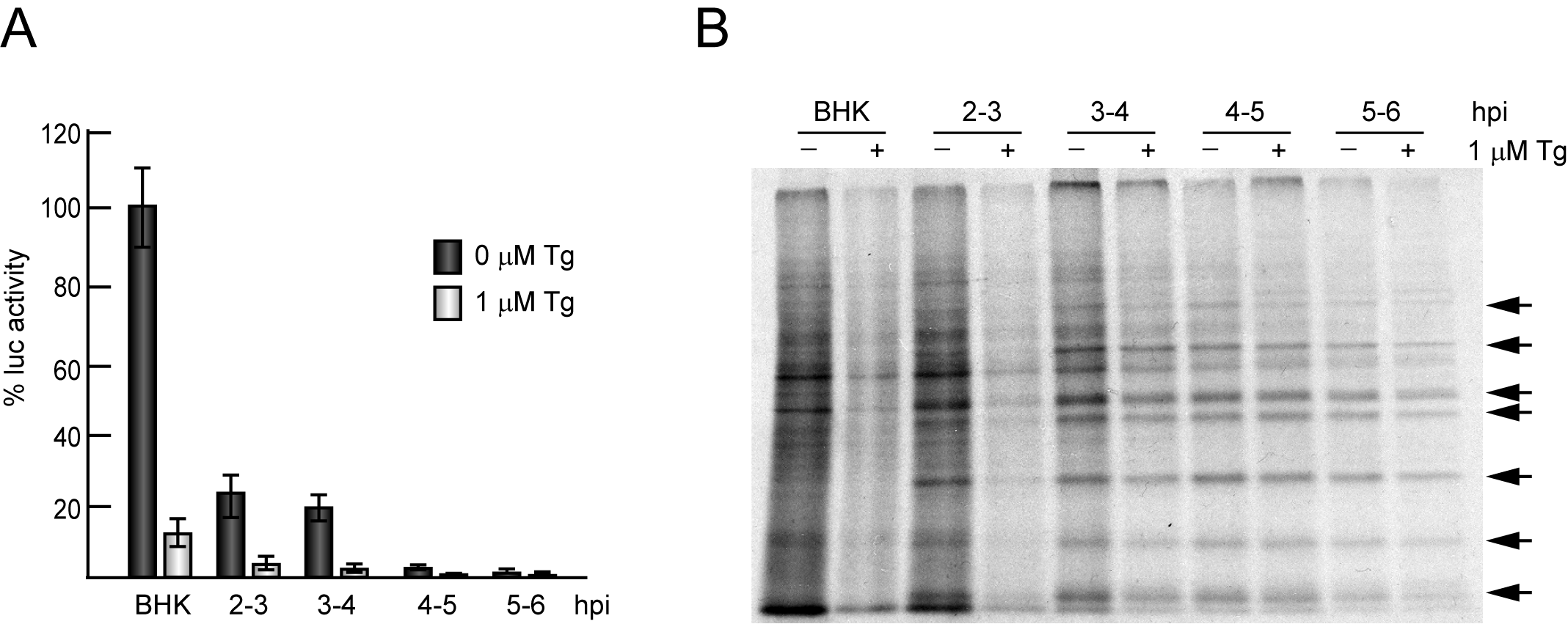

Supplement: Figure S3 — Translation of EMC-luc mRNA transfected in EMCV-infected cells. A) BHK cells were infected with EMCV (10 pfu/cell) and next transfected with EMC-luc mRNA at different times after infection. The cells were incubated for 75 min with the transcription mixture containing 5 μg EMC-luc mRNA per well of an L-24 plate in presence or absence of 1 μM Tg and then collected to measure luc activity. Luc activity values are means ± SD of three measures of the same experiment. B) Protein synthesis was analyzed in parallel. In this case the cultures were treated or not with 1 μM Tg for 15 min before adding 15 µCi of [35S]-Met, Cys per well of an L-24 plate, and continue the incubation for 1 h. The arrows indicate viral proteins. (TIF) [file pone.0022230.s003.tif]

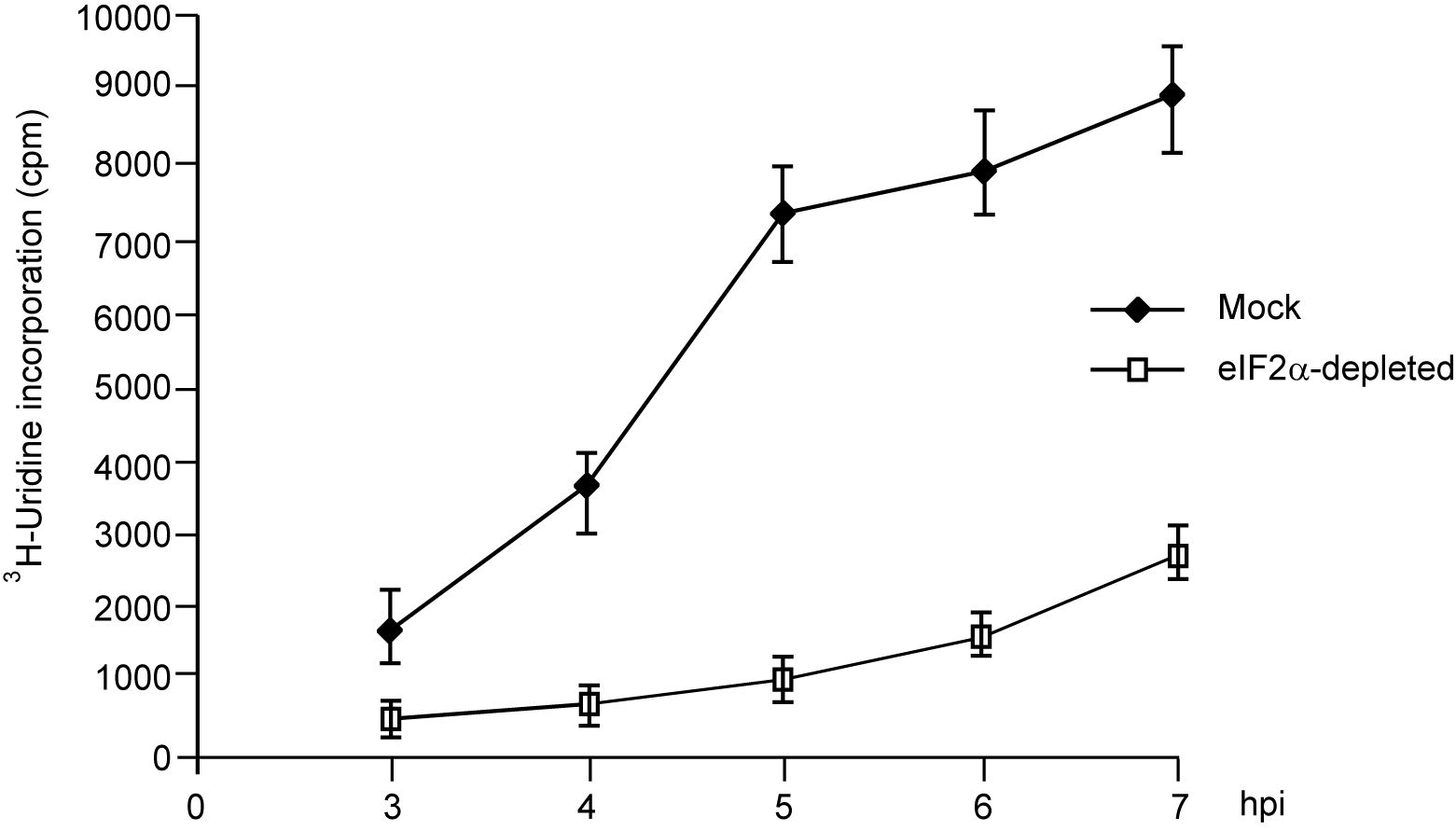

Supplement: Figure S4 — EMCV RNA synthesis in eIF2-depleted HeLa cells. Hela cells transfected with a mixture of siRNAs targeting eIF2αmRNA or mock Hela cells were infected with EMCV (10 pfu/cell) at 36 h post-transfection. Viral RNA was subsequently labeled with [3H]uridine (20 µCi/ml, final concentration) in the presence of 5 µg/ml actinomycin D. At the indicated hpi [3H]uridine incorporated was quantified in a liquid scintillation spectrometer as described before [48]. Cpm values are means ± SD of three measures of the same experiment. (TIF) [file pone.0022230.s004.tif]
